# Bio-Based Aerogels for the Removal of Heavy Metal Ions and Oils from Water: Novel Solutions for Environmental Remediation

**DOI:** 10.3390/gels10010032

**Published:** 2023-12-30

**Authors:** Antonella Caterina Boccia, Monica Neagu, Alfio Pulvirenti

**Affiliations:** 1 National Research Council, (CNR), Istituto di Scienze e Tecnologie Chimiche-SCITEC “G. Natta”, Via A. Corti, 12, 20133 Milano, Italy; alfio.pulvirenti@scitec.cnr.it; 2 Victor Babes National Institute of Pathology, 050096 Bucharest, Romania; neagu.monica@gmail.com

**Keywords:** aerogels, bio-based, oils, heavy metals, environmental remediation

## Abstract

Contamination of the aqueous environment caused by the presence of heavy metal ions and oils is a growing concern that must be addressed to reduce their detrimental impact on living organisms and safeguard the environment. Recent efficient and environmentally friendly remediation methods for the treatment of water are based on third-generation bioaerogels as emerging applications for the removal of heavy metal ions and oils from aqueous systems. The peculiarities of these materials are various, considering their high specific surface area and low density, together with a highly porous three-dimensional structure and tunable surface chemistry. This review illustrates the recent progress in aerogels developed from cellulose and chitosan as emerging materials in water treatment. The potential of aerogel-based adsorbents for wastewater treatment is reported in terms of adsorption efficacy and reusability. Despite various gaps affecting the manufacturing and production costs of aerogels that actually limit their successful implementation in the market, the research progress suggests that bio-based aerogels are ready to be used in water-treatment applications in the near future.

## 1. Introduction

Exponential urban and industrial growth has led to significant impacts on global environmental contamination and on the health of humans and all living organisms [1,2,3,4]. Environmental pollution is one of the most serious issues that need to be addressed to heal the environment, considering the cascading negative effects on air, water, and soil. Among them, water pollution is the sum of effects originating from industrial effluent and waste, municipal effluent, clinical waste, hazardous chemicals, waste from pharmaceutical and personal care products, pesticides, oils, detergents, and so on [5,6]. To solve these pressing issues, novel technologies for wastewater remediation have been developed to reduce, contain, and prevent these phenomena. Adsorption technology has been efficiently designed toward this end over the last few decades, and excellent novel adsorbent materials have been proposed to contain environmental deterioration specifically derived from heavy metal ions and oils [7,8,9,10]. The most widely used adsorbent material is activated carbon, but its use is affected by a series of costs, ranging from production to regeneration [10,11,12]. Furthermore, other adsorbent materials have been evaluated, including zeolites, clays, alumina, silica gels, polymers, and resins [13]. Emerging adsorbent materials that have gained extensive attention for environmental remediation are aerogel-based materials [14,15,16]. Aerogels are a special class of nanostructured materials showing ultra-light weight, high porosity, and tunable physicochemical properties [16,17]. Aerogels can be subdivided into three categories: inorganic, organic, and hybrid. The first category includes silica aerogels; the second includes bio-based aerogels; and the third includes hybrid materials (depending on the composition) [18].

**Figure 1:**
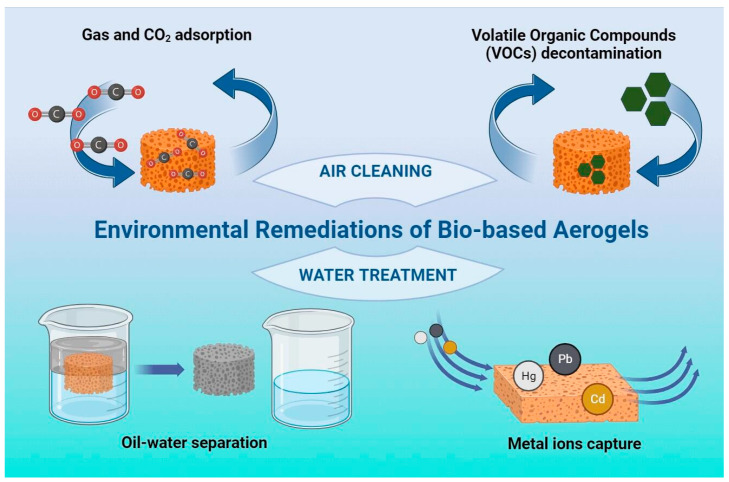
Applications of bio-based aerogels for environmental remediation.

The present review aims to discuss the more recent applications of bio-based aerogels for environmental remediation, with a special focus on the removal of heavy metal ions and oils from water solutions, elucidating, when possible, the adsorption mechanisms. In relation to their nature, bio-based aerogels from polysaccharide-based materials are environmentally friendly due to being biodegradable and biocompatible and, in particular, are less toxic and are renewable because of their natural origins, which makes them a winning factor in this challenge. Aerogels are key components in the development of new functionalized materials usable in a wide range of applications, as shown in Figure 1. Considering the great and pressing interest of the scientific community in the topic and the growing quantity of data in the literature, the present review was restricted to recent advances in aerogels from cellulose and from chitosan and the derived composite materials. Finally, this review intends to support and contribute to the expansion of these promising materials for a healthier and more sustainable world.

## 2. Results and Discussion

Aerogel is a general term referring to novel nanostructured materials characterized by very high porosity and tunable physicochemical properties that are obtained following a sol–gel process and an appropriate drying method [17,19]. Such novel materials are entering the market in everyday products and a wide portfolio of properties usable for applications in health care, foods, agriculture, energy, and environmental remediation [16]. Bio-based aerogels obtained from renewable resources and biomass are biodegradable and biocompatible due to the natural origin of the polymers, and for this reason, they greatly contribute to the sustainable concept of the bio-economy, offering promising commodities for environmental remediation.

**Figure 2:**
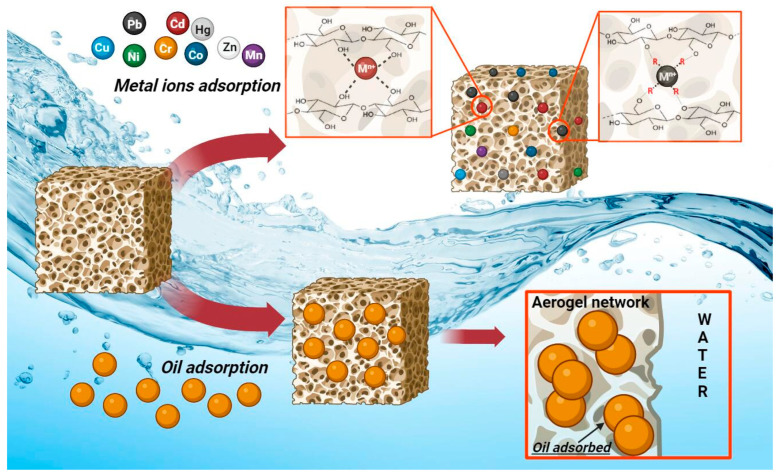
Representation of the adsorption mechanism for the removal from water of metal ions and oils.

Owing to their properties, the application of aerogels is technically appealing for the water-cleaning treatment process and usable for the adsorption of oils and the removal of heavy metal ions, as graphically shown in Figure 2.

### 2.1. Aerogels for the Removal of Heavy Metal Ions

Heavy metal pollution affects environmental health, considering that heavy metals are persistent, non-degradable, and toxic even at very low concentrations. Heavy metals can negatively impact ecosystems by contaminating the soil, the water, and also living organisms due to related bioaccumulation effects [20], making the removal of these ions of prime importance.

The literature reports several methods devoted to the removal of heavy metal ions from wastewater sources using bio-based aerogels as valid alternatives to traditional adsorbents because of their tunable physical–chemical properties, reusability, and simple regeneration processes [21,22].

Table 1 summarizes the reported literature data on the removal of some heavy metals from the aqueous phase using bio-based aerogel adsorbents.

#### 2.1.1. Cellulose-Based Aerogels

Cellulose is a natural polymer widely used for the production of aerogels because of its abundance and the possibility of modifying it by applying several different chemical processes and introducing various functional groups on the surface. Cellulose is generally modified by applying a grafting or crosslinking methodology to improve the chemical and structural stability of the produced aerogels and enrich the material surface with functional groups, such as sulfonate, carboxylate, and phosphonate groups, thus conferring a great affinity for heavy metal ions due to greater electronegative charges on the surface [23,24].

Cellulose-based aerogels modified by introducing an amine group on the surface have several applications for the removal of metal ions. Liu et al. [25] prepared aerogels by functionalizing the cellulose through a crosslinking reaction with the introduction of an amide group able to coordinate Cu(II) ions by physical adsorption and due to a functionalized surface. They found that the Cu(II) adsorption capability derives from a synergy of factors, such as the porous nature of the aerogels, together with the presence of amine and hydroxyl groups. A similar strategy was adopted for the synthesis of nanocellulose functionalized with an amine group [26], which was used for the production of aerogels for the removal of copper from water. SEM images, combined with the spectroscopic ATR-FTIR results, confirmed the role of the amine and hydroxyl groups in ion adsorption.

Aerogels based on cellulose modified with polyethyleneimine (PEI) caught the interest of several scientific groups [27,28,29,30]. Cellulose was functionalized with PEI as a crosslinker, thus allowing the introduction of a great number of amine functional groups on the surface of the cellulose, resulting in aerogels with improved chromium adsorption capability compared to native cellulose and other common adsorbents, such as activated carbon [31] or mesoporous silica modified with amino groups [32]. Two synergistic mechanisms were proposed for the adsorption of chromium: one was based on electrostatic interactions between the protonated amine groups and the chromium ions, while the second mechanism involved the redox reduction of Cr(IV) to Cr(III), balanced by the oxidation reaction of the hydroxyl and amine groups on the material surface [27]. Another paper evaluated the morphological and structural properties of synthetized nanofibrillated cellulose (NCF) after its modification by varying the amount of polyethyleneimine (PEI) [33]. The authors established the role of PEI in determining the structural stability of the final aerogels, considering that a low amount of PEI corresponds to more fragile materials compared to those obtained with increased PEI content, which were more stable and had a well-defined three-dimensional network. These materials were evaluated for removing Pb(II) from water, and they evidenced a greater adsorption capability with respect to similar adsorbent aerogels.

Aerogels from cellulose nanofibrils (CNFs)/carboxymethylcellulose (CMC) crosslinked with branched polyethylene amine (BPEI) were proposed by Mo et al. as efficient materials for the adsorption of Cu(II) from wastewater with rapid adsorption kinetics [14]. The proposed mechanism for ion capture was based on a dual effect due to the chelation of Cu(II) and ion exchange with active groups, such as hydroxyls, amines, and carboxyls. These aerogels demonstrated an excellent ability to capture metal ions even after ten sorption–desorption cycles.

Another methodology for modifying cellulose adopted the use of several acids, considering that carboxylic functional groups were found to be able to actively participate in ion capture [34,35,36]. In these works, after modifying cellulose with the introduction of carboxylic groups by adding methacrylic acid (MAA), the resulting aerogels showed interesting properties in the adsorption of several ions, such as Ni(II), Pb(II), Zn(II), and Cd(II). The results evidenced the doubled efficiency of adsorption due to the introduction of carboxylic groups on the modified NFC surface. Moreover, after grafting nanofibrillate cellulose with poly (methacrylic acid-co-maleic acid), the presence of two carboxylic groups on the aerogel surface allowed the chelation of divalent ions, as proven by FTIR spectroscopy. Following this strategy, NFC was crosslinked with polyvinyl alcohol (PVA) and acrylic acid (AA) [37], and the resulting aerogel, namely, CNFs-PVA-AA (CPA), showed a stable three-dimensional structure with a greater number of pores with a smaller size compared to the native material, responsible for the removal of metal ions from solution through coordination and ionic bond interactions. The adsorption of heavy metal ions like Cu(II) and Pb(II) was found to reach 30.0 mg/g and 131.5 mg/g when using the developed aerogels. Interestingly, there was also the possibility of reusing the aerogels for up to five cycles of use, with a maintained capacity of adsorption, which makes them good candidates for the purification of wastewater from heavy metals.

Fully bio-based aerogels (FBAs) were synthesized by combining cellulose filaments (CFs), chitosan (CS), and citric acid (CA), resulting in materials with excellent adsorption capacities for methylene blue and copper [38]. The synthetic strategy was defined as simple and scalable, thus providing aerogels with good mechanical properties due to several combined effects, such as electrostatic interactions involving CS and CFs, as well as hydrogen bonding. FBAs were finally characterized to demonstrate that the great availability of several functional groups on the aerogel surface was responsible for the excellent adsorption capacity for heavy metal ions.

Aerogels with the ability to rapidly adsorb ions, such as Cu(II), Cd(II), and Pb(II), were obtained by preparing a composite material from a cellulose nanofiber and chitosan system (CNF/CS) combined with montmorillonite activated by acid [39]. In this work, aerogels were obtained by applying a directional freezing methodology to allow an oriented porous structure, resulting in improved mechanical properties, rapid ion adsorption, and optimal reusability. Aerogels from CNFs were also modified with tannic acid (TA) based on the ability of TA to entrap heavy metal ions by chelating them [40]. These materials were able to adsorb methylene blue, a cationic dye widely used as a colorant in the textile, leather, and paper sectors. Another study proposed an eco-friendly and cost-effective synthesis of cellulose nanofibers (CNFs) grafted with cardanol-derived siloxane able to capture Cu(II) ions with an adsorption capacity of 45.6 mg/L, which was higher than those of other bio-based adsorbents [41]. In this work, the role of TA in strongly coordinating metallic ions was established to be associated with the great number of active sites on the aerogel surface due to the multi-phenolic hydroxyl groups participating in the chelation process. Moreover, the developed aerogels manifested a structure characterized by low density that makes them efficient for Cu(II) adsorption.

Aerogels from grafted nanocrystals of cellulose and acrylic acid (AA) were developed and applied for the removal of metal ions, such as Cu(II), Cd(II), and Pb(II) [42]. The highest adsorption capacities of the synthesized aerogels were found to be 872.4, 898.8, and 1026 mg/g for Cu(II), Cd(II), and Pb(II), respectively. The mechanism proposed by the authors for the adsorption of metal ions was dominated by chemisorption and by the consistency between groups of ion adsorbents, with negative charges from sulfate half-esters, carboxylic groups from AA, and hydroxyl and amine groups acting as binding sites for the metal ions.

Cellulose nanocrystals (CNCs), due to the exposed active groups on the material surface, are susceptible to modification for the production of novel binding sites, thus improving the capability of adsorbing heavy metal ions [43]. CNCs from bamboo pulp were used by Geng et al. for the production of aerogels for heavy metal ion capture. Aerogels were prepared from CNFs oxidized with TEMPO and successively modified with 3-mercaptopropyltrimethoxysilane (MPTs) to give (TO–NFC–Si–SH) as high-porosity aerogels [44]. After their modification, the aerogels showed a great number of thiol groups on the surface that were able to remove Hg(II) ions from water in a wide interval of pH values, with an adsorption capacity of 718.5 mg/g.

Aerogels designed as composites of cellulose and metal–organic frameworks (MOFs) were recently investigated for the capture of heavy metals from solutions. These materials showed excellent adsorption properties correlated with a synergic effect from their very good chemical stability and their large number of functional groups [45]. Metal–organic framework@cellulose aerogel composites were developed by Lei et al. by applying an in situ growth methodology for the production of materials able to capture Cu(II) and Pb(II) ions. The optimal performance reached was 89.40 mg/g for Pb(II); moreover, the materials were recycled for up to five cycles by cleaning with water and mostly maintained their original performance [46]. Also, for these materials, the ion adsorption process was correlated with a mechanism based on the chelation of metal ions with the functional groups on the aerogel.

In another work, hybrid cellulose-based aerogels and metal–organic frameworks were prepared by combining them with a zeolitic imidazolate framework, thus producing aerogels named ZIF-8@CA [47] with high binding potential and a high specific surface area. These hybrid aerogels showed good adsorption capacity for Cr(VI), not only from the surface of the water solution but also from the bottom, with more than 91% removal of the ions; moreover, they evidenced high hydrophobicity and a homogeneous porous structure, overcoming the previous weak properties limiting the final applications. These aerogels were able to reduce Cr(VI) ions in the water and capture heavy metal ions due to coordination with amine (-NH_2_) and carboxylic active groups (-COOH).

Carbon-based aerogels (CAs) are a class of three-dimensional porous materials with good chemical stability and excellent physicochemical properties that, together with a high surface area, superelasticity, and high-temperature resistance, enable the recyclability of the aerogels [48]. CAs have found applications for environmental remediation in removing heavy metal ions, pollutants, oils, and hazardous solvents [48,49], and recently, bio-based CAs enriched the category with the introduction of CAs developed from bio-masses based on cellulose, lignin, chitosan, and tannin or bio-masses from wastes [10].

Sodium alginate–streptomycin sulfate composite aerogel (Alg–Stre), obtained by chemical grafting, was used for the removal of heavy metal ions, such as Pb(II) and Cu(II), from water [50]. The highest observed adsorption capacities of Alg–Stre for Pb(II) and Cu(II) were considered competitive and selective with respect to a similar adsorbent and were 280 mg/g and 160.2 mg/g for Pb(II) or Cu(II), respectively. The produced materials, characterized by high specific surface area and surface energy, contributed to an extremely fast adsorption speed with excellent performance that was only slightly affected by the temperature.

A shapeable sodium alginate aerogel incorporating modified L-cysteine/UiO-67 was prepared by Du et al. and applied for the removal of Cd(II), Cu(II), and Pb(II) from water [51]. These materials were able to remove great amounts of metal ions (such as 661.2 mg/g for Pb(II), 296.2 mg/g for Cd(II), and 326.4 mg/g for Cu(II)) and to rapidly reach the equilibrium of adsorption. Also, in this case, the improved capability to capture ions was found to be due to the involvement of the functional groups in the chelation process and ion exchange with the metal ions.

Polysaccharide-based aerogels obtained from chitosan and thiourea were engineered by a series of freeze–thaw cycles and combined with formaldehyde to promote the occurrence of covalent bonds [52]. These aerogels were ultra-lightweight materials characterized by a high specific surface area and low density, with a very good capability of adsorbing Pb(II) and Ag(I) ions over five sorption–desorption cycles and very good efficiency after recycling. The selectivity of these aerogels in capturing Pb(II) and Ag(I) ions was associated with the ions’ interaction with the –NH and –S groups available on the material surface.

An et al. reported the preparation of a hybrid lignocellulose/chitosan aerogel as a probe for heavy metal detection and adsorption [53]. This material was characterized by an adsorbent skeleton, represented by a hybrid aerogel of cellulose nanofibers and chitosan; a probe for detection based on Rhodamine 6 G; and a crosslinking agent, such as polyvinyl alcohol and glutaraldehyde. This composite material demonstrated interesting hydrophilicity and the rapid capture of several metal ions, such as Hg(II), Ag(I), Al(III), Fe(III), and Cu(II), from wastewater. The developed material was found to be very structurally stable over five sorption–desorption cycles and renewable. The main reasons explaining the material adsorption performance were correlated with the synergic effects derived from a low density and high specific surface area.

A low-cost, eco-friendly calcium alginate aerogel (CAA) was prepared for the removal of lead from aqueous solutions. The results of the investigation revealed that the aerogel had high selectivity and adsorption capacity for Pb(II). The CAA was found to be able to absorb 96.4% of Pb(II) from the aqueous solution, and it could be recovered with a simple acid treatment and reused without any loss in performance [54].

A recent work reported the synthesis of hybrid mesoporous aerogels based on silica and gelatin that are able to selectively adsorb Hg(II) from water containing several heavy metals, such as Cd(II), Cu(II), Pb(I), Ag(II), Ni(II), Zn(II), and Ni(II) [55]. By changing the material composition in terms of %wt of gelatin from 4 to 24%, it was possible to increase Hg(II) removal from the water by up to 91%. Gelatin was found to be able to coordinate Hg(II) and to release it after the aerogels were washed with a solution of a complexing agent such as EDTA. Finally, the aerogels were recovered with unmodified adsorption ability for up to five cycles of continuous use.

#### 2.1.2. Chitosan-Based Aerogels

In addition to cellulose, chitosan is frequently used as a porous adsorbent material for the removal of heavy metal ions from wastewater. Chitosan is a linear polysaccharide with a structure characterized by a large number of active groups, such as amino and hydroxyl groups, enabling it to be involved in redox reactions, Van der Waals forces, hydrogen bonds, and other effects involved in the capture of metal ions. Due to the presence of active functional groups, chitosan is generally modified to improve the mechanical and chemical properties and has been successfully combined with other materials, depending on the final desired applications [56,57].

For the removal of Cu(II) from wastewater, Fan and co-workers prepared a responsive composite aerogel from chitosan and poly(acrylic acid-2-(dimethylamino)ethyl methacrylate) via physicochemical double crosslinking that was efficient, recyclable, and able to capture Cu(II) ions with an increased adsorption capacity of up to 660% relative to aerogels made from unmodified chitosan [58]. The capability to entrap Cu(II) was attributed to chelation and complexation phenomena. The adsorption capacity of the prepared composite aerogels reached 70% of the initial adsorption capacity even after six cycles.

Usually, chitosan is combined with cellulose to enhance the adsorption properties and strengthen the mechanical properties of the final aerogels. Li et al. [59] developed composite aerogels from chitosan (CS) and nanofibrillated cellulose (NFC) for the adsorption of Pb(II) from water, which showed a maximum adsorption capacity of 252.6 mg/g. Interestingly, the materials take only 5 min to reach 85% of the equilibrium adsorption capacity for Pb(II) [59], and this peculiar characteristic was attributed to the presence of oriented microchannels obtained via a directional freeze-drying approach. Gao et al. prepared an alginate/melamine/chitosan aerogel for the removal of Pb(II) from water solutions, with a maximum adsorption capability of 1331.6 mg/g at pH 5.5 [60]. The aerogels were regenerated while maintaining a good adsorption capability for up to eight sorption–desorption cycles. In this case, the authors took advantage of the free amino and aromatic nitrogen groups characterizing the melamine molecule and the useful functional groups, such as hydroxyl amino (-NH_2_), on chitosan to form complexes with metal ions and remove them from water.

Another composite aerogel was prepared from chitosan and hydroxyapatite and used for the absorption of Pb(II) from wastewater, with a capacity of up to 264.42 mg/g, while that of the porous CS material was only 5.67 mg/g. After the adsorption of Pb(II) ions, the material was regenerated via an ion exchange reaction by replacing the heavy metal ions with a high-concentration solution of Ca(II) ions [46].

High-efficient aerogels from cellulose from pineapple leaves and chitosan from shrimp waste were prepared and used for Cr(VI) removal from wastewater [61]. The work explored several freezing methodologies, including refrigeration and isotropic and anisotropic liquid nitrogen, with the aim of investigating the effects on the final structure of the synthesized aerogel-based adsorbents. The adsorption efficiency for Cr(VI) was proven, as well as the aerogel’s excellent reusability. The material behavior related to Cr(VI) adsorption strongly depends on the content of chitosan and the pH of the medium and was correlated with the electrostatic attraction between Cr(VI) oxyanions and protonated amine groups. Starting from the same raw materials, Do et al. developed ultra-lightweight composite aerogels from pineapple leaves, as a source of cellulose, functionalized with TEMPO (2,2,6,6-tetramethylpiperidinyloxy), with the introduction of carboxylate groups able to physically interact with the amino groups of chitosan, thus promoting a stable three-dimensional structure without using a crosslinker [62]. The developed composite materials showed high removal efficiencies for dyes.

Chitosan, after its modification, was also combined with polydopamine to produce aerogels (CS-PDA) using glutaraldehyde as a crosslinking agent [63]. CS-PDA exhibited excellent adsorption ability in the removal of Cr(VI) and Pb(II), with maximum adsorption capacities of 374.4 and 441.2 mg/g for Cr(VI) and Pb(II), respectively. No changes in the material performance were observed after eight sorption–desorption cycles, and the results were correlated with the surface functionalization with glutaraldehyde and with the amino and hydroxyl groups on chitosan molecular chains acting as adsorption sites for heavy metal ions. In a study by Najaflou, a cellulose sulfate/chitosan aerogel (CCA) was prepared using chitosan and cellulose sulfate and used for the removal of Pb(II) from contaminated waters. The CCA was regenerated for up to five sorption–desorption cycles with a reduction in Pb(II) removal of only 10% [64].

Recently, a green aerogel obtained from citrus peel (CP), chitosan (CS), and bentonite (BT) was prepared by Nie et al. and was found to be very efficient in the removal of Cu(II) from water solutions [65]. This aerogel, because of the abundance of active binding sites, showed an excellent Cu(II) adsorption yield of 861.58 mg/g, associated with higher selectivity. The proposed method for Cu(II) capture referred to a binding mechanism between the aerogel and metal cations through electrostatic attraction and chemical chelation between Cu(II) and the active groups on the surface of the material. The aerogel was regenerated by elution with 1 M HNO_3_, with little decrease in adsorption efficiency (5.3%) after five cycles.

### 2.2. Aerogels for Oil Removal

There are different techniques for removing oils or other organic toxic pollutants from water; these are based on (i) the physical treatment of the membrane [66]; (ii) adsorption [67]; and a chemical [68] or biological methodology [69]. Among them, the adsorbent treatment of water for the removal of oils is the most adopted method because of its efficacy and because it is more economical than the others; moreover, the used materials can be recycled for several sorption–desorption cycles.

Materials suitable for oil-spill remediation should preferably be hydrophobic, oleophilic, and eco-friendly and possess characteristics such as a high sorption capacity, stability, and recyclability. Bio-based aerogels made from polysaccharides, such as cellulose and chitosan (but also from other natural sources), are excellent materials usable for oil adsorption, as they are generally characterized by a high surface and a low density [70]. Unfortunately, they are often amphiphilic, meaning that they can absorb water and oil simultaneously. Consequentially, the hydrophobicity needs to be improved by modifying the surface wettability and the solid/liquid adhesion interaction, a process that is governed by van der Waals forces, polarity, or other physical and chemical weak forces. [71].

Table 2 summarizes the reported literature data on the removal of oils from the aqueous phase using bio-based aerogel adsorbents.

#### 2.2.1. Cellulose-Based Aerogels

Cellulose is a linear polymer formed by the interconnection of D-glucose units with 1,4-β-glycosidic bonds through an acetal function and characterized by different functional groups, like epoxies and hydroxyls, that can be modified depending on the application [72]. In nature, cellulose exists as a microfibril combination [73] and is naturally produced by plants, algae, bacteria, and some microorganisms, being the main component of their cell walls [74,75]. Although cellulose is the most abundant natural macromolecular polymer found in agricultural and forestry wastes [76], when it is used to prepare cellulose-based aerogels for oil/water separation, chemical or physical pre-treatment of the raw materials is required to obtain high-purity cellulose. To modify cellulose aerogels with low surface energy, convenient methods, such as impregnation and vapor deposition, are used, while common chemical modifiers include silanes such as triethoxysilane, castor oil siloxane, trimethylchlorosilane, methyl trimethoxysilane, PDA, and other types [77]. Corn straw was treated with sodium hydroxide and sodium hypochlorite to obtain corn straw cellulose successfully crosslinked with PVA, which, after freeze-drying and chemical vapor deposition (CVD), generated a superhydrophobic corn-straw-based aerogel [71].

Cellulose aerogels from paper wastes, such as feedstocks of cellulose, were developed through the introduction of negatively charged sulfonic acid groups on the material surface via sulfonation. Underwater superoleophobic aerogels were produced by modifying the surface of the material via a chemical vapor deposition method, introducing methyltrimethoxysilane (MTMS) and 3-decdioxypropyltrimethoxysilane. These aerogels showed excellent oil/water separation and good stability and recyclability, as demonstrated by tests based on the adsorption of 5w40 motor oil (GPTMS) [78,79].

Aerogels as polymer sponges were produced from polydimethylsiloxane, PVA, melamine, and silica, showing high mechanical properties, high adsorption capacity, and low cost, and they were usable for the separation of an efficient and recyclable mixture. In the work by Alazab [80], a polyurethane foam (PUF), as a hybrid material based on modified cellulose, was presented. The produced materials were enriched with ferric oxide to improve the chemo-physical properties; they were reusable and had a high magnetic response, and the results evidenced the achievement of a rapid sorption–desorption capability varying between 9 and 32 times its own weight, with the oil removed by simple squeezing, together with recyclability [80]. The oil–water separation ability was very good under acidic (pH = 2) and basic conditions (pH = 12).

Recently, carbon-based aerogels have been developed from carbon belts, fibers, or nanotubes, generating materials with unprecedented properties, just considering they were able to absorb oils in amounts up to 190 times their own weight [81]. Despite these excellent characteristics, these materials are affected by several drawbacks related to their non-eco-sustainable precursors and high costs, and starting from this, carbon-based aerogels were developed from vegetable raw materials from biomass [82,83].

Subsequently, cellulose-based carbon aerogels used for the separation of oil–water were introduced; they were obtained from activated carbon material via direct pyrolysis and carbonization, which are necessary processes to modulate properties such as wettability, porosity, density, elasticity, and heat resistance. To achieve better hydrophobicity and adsorption capacity, the cellulose-based carbon aerogels were modified following a pre-carbonization modification or a post-carbonization modification. The first one involves the doping of a silane reagent, micro-nanoparticles, and polymers into a cellulose dispersion to realize the in situ construction of micro-nanostructures during carbonization. In the second one, the silane reagents are mostly deposited in aerogels through vapor deposition to improve the wettability of carbon aerogels [84]. Jin et al. developed carbon aerogels (CAs) from waste newspapers as a raw carbon source by using a freeze-drying methodology and post-pyrolysis processes [85]. The developed aerogels showed good hydrophobic character and an uptake capacity for organics and oils amounting to 29–51 times their own weight.

Aerogels prepared from raw cellulose are hydrophilic; as a consequence, for the treatment of water, the starting materials have to be modified [86]. One of the adopted methods for this purpose is chemical vapor deposition for making the material hydrophobic, or alternatively, solvent exchange represents a valid alternative to modify the aerogel surface while keeping, at the same time, a hydrophilic inner surface. Other adopted methodologies for modifying raw cellulose are the deposition of substrates such as metal oxides, reduced graphene oxide, quantum dots, and nanocarbons [87].

#### 2.2.2. Chitosan-Based Aerogels

Chitosan is a natural and linear polymer consisting of glucosamine and N-acetyl-(D)-glucosamine units derived from chitin degradation by alkaline deacetylation and enzymatic degradation; it is a bio-polymer present in the exoskeletons of insects, in the cell walls of fungi (e.g., aspergillus, mucor), or in crustaceans (e.g., crabs, shrimps, prawns, lobsters) [88]. Like cellulose, chitosan is a polysaccharide widely derived from biomass and is environmentally friendly, considering its biodegradability and biocompatibility. Chitosan is extensively used in a wide range of applications because of its versatility due to the abundant presence of amine and hydroxyl groups in its structure [89]. Aerogels from chitosan and its composites are materials characterized by high specific surface area and high porosity, and they are pH-responsive due to their active functional groups; these properties make the final materials able to cover an extensive range of applications. Chitosan was modified with graphene oxide by Guo et al. [90] to produce aerogels with improved hydrophilicity and dispersibility in aqueous solutions that were able to adsorb diesel oil from seawater. A high removal efficiency of up to 95% was reached, making these materials excellent candidates for the treatment of industrial oil leakage or effluent in natural water.

Glutaraldehyde-crosslinked chitosan aerogels were obtained by Li et al. [56] as eco-friendly absorbents for wastewater over a wide range of organic solvents and oils, a treatment that, compared with conventional absorbents, displayed outstanding adsorption performance and also good reusability.

A superhydrophobic composite material was obtained from chitosan modified with sodium stearate and cellulose; it was characterized by a special structure consisting of a non-porous surface and a porous internal layer and was found to be able to separate surfactant in water–oil emulsions under gravity [91].

Ultra-light and elastic aerogels were prepared by bidirectional freezing, chemical crosslinking, and chemical vapor deposition, resulting in highly oriented wavy structures made of chitosan and cellulose nanofibrillated fibers (CNFs) for oil–water separation [92]. The idea was to obtain materials with extremely improved structural and mechanical properties, as well as recyclability, and the final results indicated that the aerogels evidenced excellent shape recovery, with a corresponding decrease in density. After 50 cycles of compression–release testing, the materials maintained about 88% of the original compressive strength and a very good adsorption capacity in several organic solvents.

A facile microbubble template technique was introduced, with the assistance of ultrasonic technology and a crosslinker, for the production of chitosan-based aerogels. The so-obtained materials were able to remove oils from wastewater and heavy metal ions, such as Cu(II) [93]. The excellent stability and reusability of the developed aerogels for the removal of oil from water have been related to the introduction of abundant microbubbles during chitosan gelation. The small microbubbles were generated in the polymeric matrix by adjusting the pH and ultrasonic treatment conditions.

Chitosan-based aerogels for recovering water from oil spills or crude-vegetable-oil–water emulsions or from highly contaminated oil-spill wastewater were prepared by Chaudhary et al. [94]. Macroporous aerogels were synthesized using agarose as a pore-forming agent and as a surface coating to create a chitosan network by crosslinking. Additionally, with this methodology, the obtained aerogels were more hydrophilic and allowed to permeate water with a purity of 99%. Moreover, they can be recovered and reused after simple compression, thus reaching a sorption–desorption capability of up to 50 cycles with a good yield.

Taking advantage of the amino groups on chitosan, a novel, low-cost, renewable aerogel was developed by reacting it with oxidized cellulose, followed by crosslinking via a Schiff base reaction and cold plasma modification, thus allowing hydrophobic modification [95]. The so-obtained material exhibited outstanding oil–water selectivity for various oils and a high absorption capacity.

### 2.3. Aerogels for the Recovery of Rare and Rare-Earth Elements

A separate section is dedicated to the possible use of aerogels for the recovery of rare and rare-earth elements (REEs) from wastewater, considering that some of them are key elements in the growing field of technologies. Their use is crucial for the production of satellites, lasers, repeaters, and generators [96], but especially in sectors dedicated to renewable energy, such as in the electrical vehicle industry and for wind turbine production [97,98]. A consequence of the expanding use of REEs for technologies is water contamination and the necessity of looking for solutions for mitigating and counteracting their environmental impacts. Therefore, aerogels represent an emerging alternative in this regard for fighting environmental pollution [99], considering that adsorption is the more efficient method for concentrating metal ions [46,100]. Additionally, aerogels should contribute to ensuring the availability of these resources.

Recently, carboxymethylated cellulose nanofibrils (CMCNFs) crosslinked with citric acid were developed by Kim et al. by applying a simple synthetic procedure, and the results evidenced high durability and good adsorption capacities for the recovery of lanthanum ((La(III)) and cerium ((Ce(III)) from aqueous solutions [98]. The absorption properties for La(III) and Ce(III) ions, exceeding 120 mg/g over five repeated cycles, were correlated with the enhanced availability of -OH and CH_3_COOH groups, which act as binding sites on the material surface, driven by electrostatic interactions among carboxylic groups on the aerogels and metal ions via ionic crosslinking.

A pioneer work was based on the use of polyurea-crosslinked calcium alginate (X-alginate) to produce aerogels for the removal, from aqueous solutions, of polyvalent metal ions, such as Eu(III) and Th(IV), with a capacity of 550 g kg^−1^ for Eu(III) and 211 g kg^−1^ for Th(IV) [101]. The sorption process was based on the formation of inner-sphere complexes between the functional groups on the surface and the cationic species of the metals. These aerogels were found to be able to completely remove Th(VI) from wastewater, while 20% of Eu(III) could be removed.

Aerogels were also developed and used for limiting uranium pollution. Uranium is an important source of nuclear energy and is widely adopted, but it can pollute the aquatic environment through a series of processes, thus affecting the health of marine organisms and, as a consequence, the food chain. For the removal of uranium from aqueous solutions, an aerogel obtained from cellulose nanofibers (CNFs) was synthesized by Wang et al. [102]. This aerogel showed excellent selectivity and regeneration ability toward uranium, with fast kinetics of adsorption. These properties are mainly due to the formation of inner-sphere surface complexes in which two –COOH groups are involved as coordinating species able to entrap UO_2_(II) ions. Due to the great hydrophilicity and surface area, together with the porous structure, these materials were able to adsorb uranium with high efficiency and limited costs.

In a recent study, the synthesis of an amidoxime-modified feather keratin aerogel was presented; this aerogel was successfully used for the removal of uranium from water even at very low concentrations (adsorption capacity of 585.88 mg·g^−1^ in an 8 ppm solution of uranium) [103]. The developed aerogel showed a porous structure and a high specific surface area that allowed it to selectively adsorb uranium from a solution in which several metal ions coexisted, and for this, it can be considered a valuable tool for extracting uranium from nuclear and seawater. Previously, Shi et al. also developed an aerogel based on poly(amidoxime) for the extraction of uranium from seawater and marine environments with a very good absorption capacity [104]. In this case, the polymer was crosslinked with chitosan, thus resulting in an aerogel with favorable physical properties and reusability for up to seven sorption–desorption cycles.

**Table 1:** Applications of bio-based aerogels for the removal of heavy metals.

Adsorbent	Modification	Heavy Metal	Adsorption Mechanism	Adsorption Performance	Specific Surface Area (m^2^/g)	Structure and Porosity	Average Pore Size (nm)	Z Potential ^a^	Ref.
Cellulose filament fibers	Crosslinking reaction with bisacrylamide	Cu(II)	Physical adsorption	51.3 mg/g		Continuously distributed large pores		From 20 mV to −10 mV (pH range: 2–10; pH_zpc_ = 4.62)	[25]
2,2,6,6-Tetramethylpiperidine-1-oxyl (TEMPO) oxidized cellulose nanofibril (TO-CNF)	Crosslinking with trimethylolpropane-tris-(2-methyl-1-aziridine) propionate (TMPTAP) and polyethyleneimine (PEI)	Cu(II)	Amino-hydroxyl group complexation	485.44 mg/g		3D multi-wall perforated cellular structure; 99.02–99.51% porosity		From 45 mV to −15 mV (pH range: 2–11; pH_zpc_ = 10.5)	[26]
Cellulose	Glutaraldehyde crosslinking reaction with polyethyleneimine (PEI)	Cr(VI)	Electrostatic interaction (–NH_3_^+^)	229.1 mg/g	36.8	High porosity (volume = 0.12 cm^3^/g)	13.5	From 9 mV to 0.5 mV (pH range: 1–8; optimal absorption pH = 2)	[27]
Cellulose nanofibrils (CNFs)/carboxymethylcellulose	Crosslinked with polyethylene amine (BPEI)	Cu(II)	Electrostatic interaction, chelation, and ion exchange with the hydroxyl, amine, and carboxyl groups	452.49 mg/g		99.56–99.35%			[14]
Cellulose/lignin from natural corncob particles	Oxidation with citric acid (CA)	Cd(II)	Ion exchange; electrostatic and π-cation interaction	1.62–42.9 mg/g					[34]
Nanocellulose (from jute)	Reaction with maleic anhydride, followed by sodium exchange of protons	Pb(II)	Chemisorption/complexation	20 mg/g, 40 mg/g, 115 mg/g,		Fused nano-whiskers; rough surface, porous and fluffy			[35]
Coconut shell biochar and tea factory waste	Introduction of carboxylic groups by adding methacrylic acid (MAA)	Cu(II), Cd(II), Pb(II),	Chelation by carboxylic and lactonic groups						[36]
Cellulose nanofiber	Crosslinked with polyvinyl alcohol (PVA) and acrylic acid (AA)	Cu(II), Pb(II)	Coordination and ionic bond interactions	30.0 mg/g (Cu); 131.5 mg/g (Pb)		Stable 3D structure, high porosity			[37]
Cellulose filament and chitosan	Crosslinked with citric acid	Cu(II)	Electrostatic interaction, hydrogen bonding	206 mg/g		High porosity			[38]
Cellulose nanofiber and chitosan	Reaction with montmorillonite activated by acid	Cu(II), Cd(II), Pb(II)		Cu(II): 181.92 mg/g; Cd(II): 163.85 mg/g; Pb(II): 170.19 mg/g		Homogeneous 3D-oriented porous structure			[39]
Cellulose nanofiber	Modification with tannic acid		Electrostatic interactions		11.821 m^2^/g	Total pore volume 0.0641 cm^3^/g	Average pore diameter of 2.538 nm		[40]
Cellulose nanofiber grafted with cardanol-derived siloxane	Modification with tannic acid	Cu(II)	Chelation	45.6 mg/L	136.41–75.66 m^2^/g	Low density, 15.53 mg/cm^3^; 3D cellulosic porous structure	5.510–3.822 nm		[41]
Cellulose nanocrystals	Grafting with acrylic acid (AA)	Cu(II), Cd(II), Pb(II)	Chemisorption and coordination by sulfate Half-esters, carboxylic (AA), hydroxyl, and amine groups	Cu(II): 872.4 mg/g; Cd(II): 898.8 mg/g; Pb(II): 1026 mg/g		Highly porous and macroporous honeycomb structure			[42]
Cellulose nanofiber	TEMPO oxidation and modification with 3-mercaptopropyltrimethoxysilane (MPTs)	Hg(II)	Coordination complex	718.5 mg/g	18.47–43.57 m^2^/g	Interconnected porous morphology, with high porosity: 99.05–99.53%			[44]
Cellulose and MOF (metal–organic framework) composites	In situ growth procedure	Cu(II), Pb(II)	Chelation	Pb(II): 81.30–89.40 mg/g; Cu(II): 31.23–39.33 mg/g	1251.3 m^2^/g; 800.9 m^2^/g	Surface with many micron-sized macropores (porosity: 90.5%; 84.6%)			[46]
Cellulose and MOF (metal–organic framework)	Combination with zeolitic imidazolate framework	Cr(VI)	Metal ion reduction and capture by the amine (NH_2_) and carboxylic active groups (-COOH)	41.8 mg/g		Homogeneous, highly porous structure with zeolitic imidazolate framework-8 (ZIF-8)			[47]
Lignocellulose/chitosan	Crosslinked with polyvinyl alcohol and glutaraldehyde	Hg(II), Ag(I), Al(III), Fe(III), Cu(II)	Rhodamine-6G as detection probe			Low density and high specific surface area			[53]
Chitosan	Physicochemical double crosslinking with poly(acrylic acid-2-(dimethylamino)ethyl methacrylate)	Cu(II)	Chelation and complexation	660%	3.42–5.94 m^2^/g	Rough and porous structure, with super-low density (0.043 g/cm^3^), pore volumes of 0.005 and 0.009 cm^3^ g^−1^	Mean pore sizes: 6.33 and 7.41 nm	(pH_pzc_ = 9.83)	[58]
Chitosan	Composite with nanofibrillated cellulose (NFC)	Pb(II)	Interaction with amino groups	252.6 mg/g		Highly oriented microchannel structure			[59]
Chitosan	Alginate/melamine composite	Pb(II)	Complexation by amino and aromatic nitrogen groups	1331.6 mg/g		3D network structures, with heterogeneous pores			[60]
Chitosan (shrimp waste)	Composite with cellulose (pineapple leaves)	Cr(VI)	Electrostatic interaction between Cr(VI) oxyanions and protonated amine groups	210.6–211.4 mg/g		Ultra-lightweight (20–30 mg/cm^3^), highly porous (above 97.5%)			[61]
Modified chitosan	Combination with polydopamine and crosslinking with glutaraldehyde	Cr(VI), Pb(II)	Electrostatic attraction or chelation	Cr(VI): 374.4 mg/g; Pb(II): 441.2 mg/g	77.3 m^2^/g	Perfect three-dimensional network structure, large pores	24.9 nm	From 30/20 mV to −5/−10 mV (pH range: 2–8; pH_pzc_ about 5.6 and 7.5)	[63]
Chitosan	Cellulose sulfate combination	Pb(II)	Ion interaction with functional groups	197.1 mg/g	0.786 m^2^/g	Total pore volume of 6.44 × 10^−3^ cm^3^/g)	Mean pore diameter of 32.77 nm		[64]
Chitosan	Combination with citrus peel (CP) and bentonite (BT)	Cu(II)	Electrostatic interaction and chelation	861.58 mg/g	41.28, 48.36, and 46.57 m^2^/g	More ductile, dense, and compact three-dimensional porous structure (presence of abundant pores)	5–25 μm	Between 20 mV and −25 mV (pH range: 1.5–5.5; pH_pzc_: 2.10, 3.22 and 3.71)	[65]
Chitosan/thiourea	Combination with formaldehyde	Pb(II), Ag(I)	Ion interaction with –NH and –S groups		416.64–447.26 m^2^/g	High specific surface area and low density			[52]
Sodium alginate–streptomycin sulfate composite aerogel (Alg–Stre)	Chemical grafting	Pb(II), Cu(II)	Chelation process and ion exchange	280 mg/g and 160.2 mg/g for Pb(II) or Cu(II)		High specific surface area and surface energy			[50]
Sodium alginate aerogel	Incorporation of modified L-cysteine/UiO-67	Cd(II), Cu(II), Pb(II)	Chelation process and ion exchange	661.2 mg/g for Pb(II); 296.2 mg/g for Cd(II); 326.4 mg/g for Cu(II)	337.66–1037.14 m^2^/g				[51]
Mesoporous silica/gelatin-based aerogel		Hg(II)	Metal complex coordination by gelatin	209 mg/g		Hybrid backbones built from spherical blocks (globules)	From 32 to 17 nm	From −22 mV to −14 mV at pH = 4; from −27 mV to −23 mV at pH = 6	[55]

(^a^) Parameter used to determine the net surface charges of adsorbents that develop from the protonation and deprotonation of surface functional groups.

**Table 2:** Applications of bio-based aerogels for oil removal.

Adsorbent	Modification Method	Absorbed Oil	Performance	Specific Surface Area (m^2^/g)	Structure and Porosity	Average Pore Size (nm)	Ref.
Cellulose nanofibers (CNFs)	Methyltrimethoxysilane and 3-glycidoxypropyltrimethoxysilane modification	Toluene	56 g/g	3.9 m^2^/g	3D open-cell geometry interconnected with minor cellular pores, thin sheets, nanoparticles, and nanofilaments; high pore volume (0.06 m^3^/g)	Macropores (>50 nm), mesopores (2–50 nm), and micropores (<2 nm)	[78]
Recycled cellulose fibers	Kymene crosslinking, methyltrimethoxysilane (MTMS) coating	5w40 motor oil	95 g/g		Highly porous 3D structures (porosity: 97.2–99.4%)	Macropores (>50 nm)	[79]
Modified cellulose/polyurethane foam (PUF)	Fe_3_O_4_ NP enrichment	n-Hexane	>97.68%		Highly porous interconnected 3D structure		[80]
Cellulose from bamboo powder	Composite formation with graphene oxide; crosslinking agent: epichlorohydrin (ECH)	Methylene blue (MB); tetracycline (TC)	MB: 421.9 mg/g TC: 163.4 mg/g	173 m^2^/g	3D network structure, fluffy and porous architectures	3.6 nm	[87]
Cellulose nanofiber (CNF)	Modification with deep eutectic solvents; carbonization	Acetone, n-hexane, methanol, sesame oil, paraffin oil, decane, gasoline, diesel, phenoxin, chloroform, toluene, ether, pump oil, acetic ether, oleic acid	From 74% to 95%		Ordered lamellar structure, rough surface; many macropores	Micro- and mesoporous features: broad pore size distributions (from 0 to 50 nm)	[84]
Cellulose from waste biomass	Post-pyrolysis	Series of oils and organics (ethanol, methanol, acetone, chloroform, ethyl acetate, benzene and methylbenzene, gasoline, cooking oil, olive oil, and pump oil)	pump oil 63.9%, ethanol 97.0%, gasoline 100%		Porous and interconnected 3D networks		[85]
Chitosan	Graphene oxide modification; glutaraldehyde crosslinking	Diesel oil	12.56–12.03 g/g;	641.6 m^2^/g; 530.3 m^2^/g; and 606.4 m^2^/g	Interconnected, mesoporous, three-dimensional network		[90]
Chitosan	Glutaraldehyde crosslinking	Crude oil, diesel	1.07 g/g, 31.07 g/g		Porous structure, low density (0.0283 g/cm^3^), high porosity (97.98%)		[56]
Chitosan	Sodium stearate and cellulose	Surfactant in water–oil emulsions			Non-porous surface and porous internal layer (density and porosity of 0.065 g/cm^3^ and 95%, respectively)		[91]
Chitosan/cellulose nanofibrillated fiber (CNF)	Crosslinking and CVD	Several organic solvents	more than 100 g/g; chloroform up to 232 g/g		Highly oriented wavy structures		[92]
Chitosan	Microbubble template technique/ultrasonic technology/crosslinking	Hexane, cyclohexane, vacuum pump oil, silicone oil, and soybean oil	separation efficiency > 91%		3D interconnected porous architectures, porosity of 97.6%		[93]
Chitosan	Bio-origin genipin as crosslinker	Oil spill, crude vegetable oil	Efficiency of 99% pure water recovered		Highly porous structure, large pore size	Macroporous range of 40–50 μm	[94]
Chitosan	Oxidized cellulose; crosslinking and cold plasma modification	Selectivity for various oils	13.77–28.20 g/g		Macroporous structures in 3D network structures with high porosity (96.44%, 96.79%, and 96.81%)		[95]

## 3. Conclusions and Remarks

Recent bio-based aerogel adsorbents have been illustrated as green, efficient, and sustainable materials for the treatment of water by removing heavy metal ions and oils that are toxic to the aqueous environment and living organisms. The presented bioaerogels showed great affinity for various pollutants and excellent sorption–desorption capacity and regenerability, but efforts are necessary for scaling up the use of these materials from the laboratory scale to real water treatment, which actually limits their effective applications and expansion in the market. Therefore, from the perspective of safeguarding human health and the environment, the possible secondary pollution eventually coming from aerogels post-treatment and post-regeneration, as well as from the end-of-life of the materials, should be avoided. Challenges to be addressed in the near future by the scientific community working on bioaerogels should be considered to fill the gap in the use of aerogels in real water systems and, consequently, to evaluate the potential toxicity derived from their dispersion in the environment.

## Data Availability

The data presented in this study are available in the article.
